# Intrahepatic Cholestasis in Sickle Cell Disease: A Case Report

**DOI:** 10.1155/2011/975731

**Published:** 2010-12-21

**Authors:** Denise Menezes Brunetta, Ana Cristina Silva-Pinto, Maria do Carmo Favarin de Macedo, Sarah Cristina Bassi, Joao Victor Piccolo Feliciano, Fernanda Borges Ribeiro, Benedito de Pina Almeida Prado, Gil Cunha De Santis, Ivan de Lucena Angulo, Dimas Tadeu Covas

**Affiliations:** Hematology Division and Center for Cell Based Therapy, Department of Internal Medicine, Medical School of Ribeirao Preto, University of Sao Paulo, 14051-140 Ribeirao Preto, SP, Brazil

## Abstract

Intrahepatic cholestasis (SCIC) is an uncommon but potentially fatal complication of sickle cell disease (SCD), with a high death rate, observed mainly in patients with homozygous sickle cell anemia. Herein, we describe a case of severe SCIC treated successfully with aggressive manual exchange transfusion (ET). The patient was admitted with enlarged liver and signs of hepatic failure, such as hyperbilirubinemia and coagulopathy. There was no evidence of viral hepatitis or biliary obstruction. We performed several sessions of ET in order to reduce the percentage of HbS to levels inferior to 30%, which was successfully accomplished. The patient had a complete recovery of hepatic function. This case has shown that ET is an effective treatment of SCIC and should be introduced early on the onset of this severe complication.

## 1. Introduction

Acute sickle hepatic crisis, also known as intrahepatic cholestasis (SCIC), is an uncommon but potentially fatal complication of sickle cell disease (SCD), with a high death rate, observed mainly in patients with homozygous sickle cell anemia. Its presentation ranges from a benign hyperbilirubinemia to a fulminant hepatic failure [1–3]. This syndrome is characterized by abdominal pain in the upper right quadrant, enlarged liver, acholic or light stools, marked hyperbilirubinemia, and variable elevation of aminotransferases (AST and ALT) levels, but usually no more than 1–3 times normal [4–6]. In more severe cases, renal dysfunction and alteration of coagulation tests can be observed [3].The diagnosis requires exclusion of viral hepatitis and cholecystitis/choletithiasis. Treatment patterns are not well established and range from supportive care to exchange transfusion. However, early recognition of severe cases and aggressive exchange transfusion of red blood cells (RBCs) are considered crucial to attain a favorable outcome [7–10]. Herein, we have reported a case of severe SCIC with a favorable outcome after aggressive exchange transfusion of RBC.

## 2. Case

**Figure 1:**
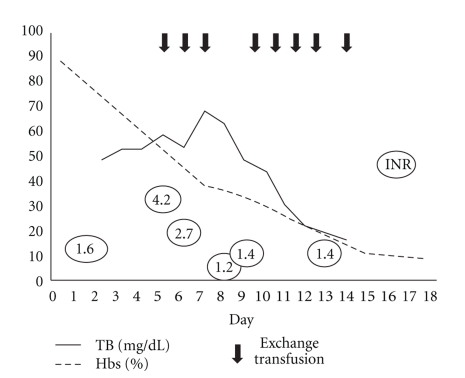
Exchange transfusion and evolution of bilirubin levels, hemoglobin S (HbS) percentage and INR according to the day of hospital admission.

A 41-year-old male patient with sickle cell anemia, and no family history of SCD, was admitted to the emergency ward of our institution due to upper right quadrant pain in abdomen started a few hours before. Moreover, the patient noted worsening of jaundice, choluria, and fatigue. Physical exam demonstrated jaundice, right upper quadrant tenderness, an enlarged liver as well as a distended abdomen. The patient denied fever, diarrhea, or bleeding. There were no signs of encephalopathy. He had a history of recurrent pain crisis and was submitted to cholecystectomy a few years before because of the presence of gallstones. The abdominal ultrasonography showed hepatomegaly with signs suggestive of chronic liver disease, a moderate ascitis, and absence of gallbladder and of spleen. There were no evidences of dilated extrahepatic biliary ducts or obstruction. The laboratory data on admission were total bilirubin 39 mg/dL (direct bilirubin 21.4 mg/dL), hemoglobin concentration (Hb) 6.0 g/dL, hematocrit (Hct) 18%, white blood cell (WBC) count 23,100/*μ*L, platelet count 317,000/*μ*L, alanine aminotransferase (ALT) 76 U/L, aspartate aminotransferase (AST) 210 U/L, alkaline phosphatase 557 U/L, gama-glutamyltransferase (gama-GT) 127 U/L, albumin 2.8 g/dL, INR 1.6 and partial thromboplastin time (PTT) 162,2 s (ratio 6,19). The serology tests for hepatitis B and C and for HIV were negative. The patient was submitted to aggressive manual exchange transfusion of RBC, in order to reduce the percentage of HbS to levels inferior to 30%. Thirty milligrams of vitamin K were administered on the first day with no recovery of INR or PTT. Simple transfusion of fresh frozen plasma was performed whenever the INR values were higher than 2.0. Evolution of bilirubin levels, hemoglobin S (HbS) percentage and INR is shown in Figure 1 and Table 1.

## 3. Discussion

In this paper we report one case of intrahepatic cholestasis, which is a rare but potentially fatal complication of sickle cell disease. Its etiology remains unknown, but it is believed that the deformed erythrocytes adhere to the hepatic vascular endothelium resulting in sludging and congestion of vascular beds, followed by tissue ischemia, infarction, and liver dysfunction in the more severe cases. Moreover, SCIC can superimpose on a liver with chronic disease due to hemochromatosis and hepatitis.

 Ahn and colleagues [11] reported 7 cases from their institution and another 37 cases from the literature published between 1953 and 2002. The authors classified the patients in 2 groups according to disease severity. Group I patients presented a total bilirubin level of 27.6 mg/dL and no evidence of severe hepatic dysfunction, whereas patients from group II presented a mean bilirubin of 76.8 mg/dL and signs of severe hepatic dysfunction, manifested by prolonged coagulation tests (PT and PTT) and/or encephalopathy. Overall mortality was 4% in group I and 64% in group II. However, only 2 of 9 who were submitted to RBC exchange transfusion died, whereas 12 of 13 who did not receive transfusion died. This result indicates that exchange transfusion is the basis of SCIC treatment as suggested by previous studies [12–14]. In the case described here the diagnosis of SCIC was suspected because the sudden hyperbilirubinemia associated with a modest elevation of aminotransferases and severe liver dysfunction manifested by coagulopathy. Viral hepatitis can present a similar clinical feature, however elevated transferases is a frequent finding, sometimes with values higher than 1000 U/L. Furthermore, positive serology helps to define viral hepatitis. Another potentially confounding hepatic condition is hepatic sequestration that present tender hepatomegaly associated with decrease in Hb concentration and reticulocytosis, a mild to moderate increase in amino transferases, but not extreme hyperbilirubinemia. The so-called hepatic crisis is another hepatic condition considered in the differential diagnosis of SCIC. It is characterized by tender hepatomegaly, increasing jaundice, a mild to moderate elevation of amino transferases, but the bilirubin concentration seldom exceeds 13 mg/dL. The case described here presented clinical features suggestive of SCIC, and we performed aggressive manual RBC exchange transfusions aiming a decrease of HbS to levels below 30%, which was successfully accomplished. The patient presented a favorable evolution, and his hepatic function recovered to the previous pattern. Currently, the patient is maintained in a scheme of chronic RBC transfusion. This case reinforces the need of performing ET for intrahepatic cholestasis treatment and should be initiated promptly.

## Figures and Tables

**Table 1:** Evolution of bilirubin, HbS percentage, and INR with exchange transfusion (ET).

	D1 of ET	D5 of ET	D10 of ET
Bilirubin level (mg/dL)	58.9	50.19	16.99
HbS percentage	88.40	34.20	9.75
INR	4.21	1.4	1.4
